# Progressively De-Differentiated Pancreatic Cancer Cells Shift from Glycolysis to Oxidative Metabolism and Gain a Quiescent Stem State

**DOI:** 10.3390/cells9071572

**Published:** 2020-06-28

**Authors:** Giulia Ambrosini, Elisa Dalla Pozza, Giuseppina Fanelli, Claudia Di Carlo, Andrea Vettori, Giuseppe Cannino, Chiara Cavallini, Cristian Andres Carmona-Carmona, Jessica Brandi, Sara Rinalducci, Maria Teresa Scupoli, Andrea Rasola, Daniela Cecconi, Marta Palmieri, Ilaria Dando

**Affiliations:** 1 Department of Neurosciences, Biomedicine and Movement Sciences, University of Verona, 37134 Verona, Italy; giulia.ambrosini@univr.it (G.A.); elisa.dallapozza@univr.it (E.D.P.); cristianandres.carmonacarmona@univr.it (C.A.C.-C.); mariateresa.scupoli@univr.it (M.T.S.); marta.palmieri@univr.it (M.P.); 2 Department of Ecological and Biological Sciences, University of Tuscia, 01100 Viterbo, Italy; giuseppina.fane@gmail.com (G.F.); sara.r@unitus.it (S.R.); 3 Department of Biotechnology, University of Verona, 37134 Verona, Italy; claudia.dicarlo@univr.it (C.D.C.); andrea.vettori@univr.it (A.V.); jessica.brandi@univr.it (J.B.); daniela.cecconi@univr.it (D.C.); 4 Department of Biomedical Sciences, University of Padova, 35122 Padova, Italy; gcann1@libero.it (G.C.); andrea.rasola@unipd.it (A.R.); 5 Research Center LURM (Interdepartmental Laboratory of Medical Research), University of Verona, 37134 Verona, Italy; chiara.cavallini@univr.it

**Keywords:** cancer stem cells, pancreatic ductal adenocarcinoma, cancer metabolism, quiescence, metabolic plasticity

## Abstract

Pancreatic ductal adenocarcinoma (PDAC) is typically characterized by high chemoresistance and metastatic spread, features mainly attributable to cancer stem cells (CSCs). It is of central interest the characterization of CSCs and, in particular, the study of their metabolic features in order to selectively identify their peculiarities for an efficient therapeutic approach. In this study, CSCs have been obtained by culturing different PDAC cell lines with a specific growth medium. Cells were characterized for the typical stem/mesenchymal properties at short-, medium-, and long-term culture. Metabolomics, proteomics, analysis of oxygen consumption rate in live cells, and the effect of the inhibition of lactate transporter on cell proliferation have been performed to delineate the metabolism of CSCs. We show that gradually de-differentiated pancreatic cancer cells progressively increase the expression of both stem and epithelial-to-mesenchymal transition markers, shift their metabolism from a glycolytic to an oxidative one, and lastly gain a quiescent state. These quiescent stem cells are characterized by high chemo-resistance, clonogenic ability, and metastatic potential. Re-differentiation reverts these features, re-activating their proliferative capacity and glycolytic metabolism, which generally correlates with high aggressiveness. These observations add an important piece of knowledge to the comprehension of the biology of CSCs, whose metabolic plasticity could be exploited for the generation of promising and selective therapeutic approaches for PDAC patients.

## 1. Background

Pancreatic ductal adenocarcinoma (PDAC) is the fourth leading cause of cancer-related death and is expected to become the second within the next decade [1]. At the time of diagnosis, most patients have an unresectable tumor and, among patients who undergo surgical resection, more than 85% with no evidence of metastasis die within 5 years [2,3], consistently with early spread. Although the understanding of pancreatic cancer biology and genetics has improved, no significant advances in PDAC treatment have been realized in more than 10 years [4], especially due to high chemotherapy and radiation resistance [5]. An increasing number of studies are showing that these traits of PDAC are due to the presence of a small subset of cells with very aggressive features, called cancer stem cells (CSCs), which are responsible to drive tumorigenesis and to play a fundamental role in disease relapse [6,7]. Interestingly, in a mouse model of PDAC, it has been shown that cellular dissemination leading to metastasis occurs before the formation of an identifiable primary tumor [8]. This behavior has been associated with the presence of circulating pancreatic cells that show a mesenchymal phenotype and express typical stem markers, thus linking CSCs to epithelial-to-mesenchymal transition (EMT) [8]. Indeed, it is widely accepted that CSCs and EMT are strictly interconnected and that activation of the EMT program is necessary not only for the physical dissemination of tumor cells to distant tissues, but also for their entrance into the CSC state [9]. Furthermore, in order to proliferate and to colonize a secondary organ, extravasated mesenchymal cancer cells need to perform the opposite transformation, i.e., the mesenchymal-to-epithelial transition (MET) [9]. This interchangeability of state highlights the crucial roles of CSCs in carcinogenesis, metastasis, relapse, and resistance to standard anti-neoplastic therapies [10,11]. Therefore, the study of CSC features is crucial for their targeting, which may permanently remove tumor and avoid late relapse [12].

In addition, CSCs possess particular metabolic properties that distinguish them from the bulk of the tumor. Importantly, the metabolic requirements of cancer cell subpopulations can be exploited for therapeutic purposes [13], and thus the study of CSC metabolism may open the way for the discovery of new potential drugs. Consistently, Viale et al. have found that dormant tumor cells with CSC properties and oxidative metabolism survive Kras ablation in a mouse model of pancreatic cancer and are responsible for tumor relapse [14]. However, metabolic profiling of CSCs has generally revealed inconsistent phenotypes and it is not yet clear whether they preferentially rely on a more glycolytic or oxidative metabolism in comparison to differentiated tumor cells [15,16]. This divergence may be attributable to the different protocols used to obtain or isolate CSCs. Ideally, their isolation directly from in vivo tumors appears to be the best approach for their metabolic characterization. However, this procedure has two main limits: First, the low CSC abundance in the tumor mass renders necessary their in vitro amplification to get enough material to perform biochemical experiments [17], and second, the choice of CSC specific surface markers for their sorting from the tumor bulk may lead to the loss of CSC subtypes that do not express the chosen markers [18]. For these reasons, cancer cell lines remain an indispensable tool for CSC research [6,19]. Up to now, studies of CSC metabolism have generally focused on short term culture. We have previously shown that CSCs derived from the PDAC cell line Panc1 cultured for short periods preferentially rely on glycolysis, fatty acid synthesis, and mevalonate pathways [20]. The metabolic changes and properties of CSC over longer time periods, which may more accurately reflect their in vivo state, is unexplored.

In this study, we have progressively de-differentiated PDAC cells and followed their metabolic needs towards the acquisition of the final stem/mesenchymal state. We have characterized both stem and metabolic properties of CSCs at three different de-differentiation stages and we demonstrate that, instead of possessing static metabolic features, gradually de-differentiated CSCs show a metabolic plasticity that ends with the acquisition of a quiescent state. These dormant cells can re-emerge from quiescence, re-start to proliferate, and re-activate metabolic requirements for lactate accumulation, a sign of high aggressiveness. Our findings have important implications for the metabolic targeting of CSCs for therapy and suggest that metabolic plasticity may represent an obstacle for the use of metabolic inhibitors.

## 2. Materials and Methods

### 2.1. Drugs and Chemicals

Gemcitabine (Jemta, Sandoz, USA) and Oxaliplatin (Sigma-Aldrich, Germany) were solubilized in water. Sorafenib, (BAY43-9006, Bayer) and AZD3965 (Cayman Chemical Company) were solubilized in DMSO. Gemcitabine was stored at −80 °C, Oxaliplatin at +4 °C, Sorafenib at room temperature until use, and AZD3965 at −20 °C. Vybrant™ Dil cell-labeling solution (Invitrogen, USA) was stored at +4 °C.

### 2.2. Cell Lines

The pancreatic adenocarcinoma cell lines Panc1, PaCa44, PaCa3, and PC1J were grown in RPMI-1640 supplemented with 10% FBS and 50μg/mL gentamicin sulfate (all from Gibco/Life Technologies, USA), here reported as differentiated-cell medium (DM), and were maintained at 37 °C with 5% CO_2_. CSCs were obtained as previously described [6]. Briefly, adherent cells were washed twice in 1× PBS (Gibco/Life Technologies, USA) and then cultured in stem-specific medium (SsM), i.e., DMEM/F-12 without glucose (US Biological Life Sciences, USA) supplemented with 1 g/L glucose, B27, 1 μg/mL fungizone, 1% penicillin/streptomycin (all from Gibco/Life Technologies, USA), 5 μg/mL heparin (Sigma/Merck), 20 ng/mL epidermal growth factor (EGF), and 20 ng/mL fibroblast growth factor (FGF) (both from PeproTech, United Kingdom). Panc1, PaCa44, PaCa3, and PC1J CSCs were cultured in flasks with a hydrophobic surface specifically designated for the growth of suspension cells and were maintained at 37 °C with 5% CO_2_ in the SsM until 8–12 weeks, refreshing twice a week with new medium. Before each experiment, the cells were passed through a cell stranier (40 μm) to separate and maintain only the cell aggregates/spheres, which were trypsinized to obtain single cell suspension. The viability percentage for cell aggregates with a size >40 μm was always higher than 85% (see “Cell proliferation assay” chapter for the method). In particular, the average of the percentage of viability for Panc1 cell line with a size >40 μm was 94% for parental (P) cells, 93% for CSCs after 2 weeks, 89% for CSCs after 4 weeks, 89% for CSCs after 8 weeks of culture in SsM. Adherent cells derived from CSCs (AdCSCs) were obtained by completely replacing the SsM, in which CSCs were growing, with the DM. In these conditions, cells were cultured for more than 2 months. Bright field cell images were acquired with inverted microscope (Axio Vert. A1, Zeiss, Germany). In all the assays, both P cells and AdCSCs were cultured in proliferating conditions and not in cell growth arrest due to contact inhibition.

### 2.3. RNA Extraction and qPCR

Total RNA was extracted from 1 × 10^6^ cells (Panc1, PaCa44, PaCa3, and PC1J parental cells and their respective CSCs) using TRIzol Reagent (Life Technologies, USA) and 1 μg of RNA was reverse transcribed using first-strand cDNA synthesis. Real-time quantification was performed in triplicate samples by SYBR-Green detection chemistry with GoTaq qPCR Master Mix (Promega, USA) on a QuantStudio 3 Real-Time PCR System (Thermo Fisher Scientific, USA). The primers used were: CDH1 forward, 5′-GACACCAACGATAATCCTCCGA-3′ and reverse, 5’-GGCACCTGACCCTTGTACGT-3′; CyclinB1 forward, 5′-CATGGTGCACTTTCCTCCTT-3′ and reverse, 5′-AGGTAATGTTGTAGAGTTGGTGTCC-3′; Enolase forward, 5′-TACGTTCACCTCGGTGTCTG-3′ and reverse, 5′-TCTCCCTGGCATGGATCTT-3′; GLUT1 forward, 5′-CATCATCTTCATCCCGGC-3′ and reverse, 5′-CTCCTCGTTGCGGTTGAT-3′; MAD2L1 forward, 5′-TTGCTTGTAACTACTGATCTTGAGC-3′ and reverse, 5′-TTCTGAACTGAACACTTGTATAACCA-3′; NANOG forward, 5′-AGTCCCAAAGGCAAACAACCCACTTC-3′ and reverse, 5′-TGCTGGAGGCTGAGGTATTTCTGTCTC-3′; OCT3/4 forward, 5′-GACAGGGGGAGGGGAGGAGCTAGG-3′ and reverse, 5′-CTTCCCTCCAACCAGTTGCCCCAAAC-3′; PFK forward, 5′-TGTGGTTCGAGTTGGTATCTT-3′ and reverse, 5′-GCACTTCCAATCACCGTGCC-3′; RPLP0 forward, 5′-ACATGTTGCTGGCCAATAAGGT-3′ and reverse, 5′-CCTAAAGCCTGGAAAAAGGAGG-3′; SOX2 forward, 5′-GGGAAATGGGAGGGGTGCAAAAGAGG-3′ and reverse, 5′-TTGCGTGAGTGTGGATGGGATTGGTG-3′; ZEB1 forward, 5′-GTTACCAGGGAGGAGCAGTGAAA-3′ and reverse, 5′-GACAGCAGTGTCTTGTTGTTGTAGAAA-3′; and housekeeping gene SDHA forward, 5′-GGACCTGGTTGTCTTTGGTC-3′ and reverse, 5′-CCAGCGTTTGGTTTAATTGG-3′ [21].

The cycling conditions used were: 95 °C for 10 min, 40 cycles at 95 °C for 15 s, 60 °C for 1 min, 95 °C for 15 s, and 60 °C for 15 s. The average of cycle threshold of each triplicate was analyzed according to the 2-ΔΔCt method. The heatmap, also called clustergram, is a graphic representation of the unsupervised hierarchical clustering of target gene expression across all samples in the study. The software, which is the Relative Quantification application of Thermo Fisher Connect, uses Pearson’s correlation to calculate distances between samples and assays for hierarchical clustering based on their ΔCT values.

### 2.4. Cell Proliferation Assay

Panc1 parental cells and CSCs at 2, 4, and 8 weeks of culture were plated in 96-well cell culture plates (5 × 10^3^ cells/well) in their respective medium and were incubated at 37 °C with 5% CO_2_. Viable cells were counted by Trypan Blue dye exclusion after 2, 4, 7, and 10 days of culture. The doubling time was calculated using the formula T = (T_2_−T_1_) × log2/log (Q_2_/Q_1_), where: T_1_, day 0; T_2_, day 10; Q_1_, cell number at day 0; and Q_2_, cell number at day 10. To test the cell viability after treatment, cells were seeded in 96-well plates (5 × 10^3^ cells/well) and, the day after, were incubated with compounds at the following concentrations: 50 nM AZD3965, 100 µM Oxaliplatin, 50 µM Gemcitabine, and 20 µM Sorafenib. At the end of the treatment (72 h for AZD3965 and 48 h for all other compounds), cell growth was measured by Resazurin assay (Immunological Sciences, Italy) according to the manufacturer’s protocol and fluorescence was measured by Tecan GENios Pro Microplate reader (Ex: 535 nm, Em: 590 nm).

### 2.5. Clonogenic Assay

Briefly, 1 × 10^6^ Panc1 parental cells and CSCs at 2, 4, and 8 weeks of culture were washed with 1X PBS and incubated with CellTrace™ CFSE (1:1000) (Thermo Fisher Scientific, USA) at 37 °C for 10 min, protected from light. Cold culture medium was added to block the reaction and the cells were incubated for 5 min in ice. After 2 washes with 1X PBS, the green cells (Ex: 488 nm, Em: 530/30 nm) were sorted and 1 cell/well was plated in DM for parental cells and in SsM for CSCs in 96-well cell culture plates with a FACSAria cell sorter equipped with a 100 μm nozzle (Beckton Dickinson, USA). At least three 96-well plates for each condition were prepared. The wells in which the cells were more than 3, indicating the ability of a single cell to duplicate, were counted after 2 weeks. The cells were photographed by inverted microscope (Axio Vert. A1, Zeiss, Germany) and the diameter of the spheres was measured after 1 month with ImageJ software (ImageJ, http://rsb.info.nih.gov/ij/, 1997–2008).

### 2.6. Soft Agar Colony Formation Assay

Panc1 parental cells and CSCs at 2, 4, and 8 weeks of culture were resuspended in 2X RPMI-1640 medium or 2X DMEM/F-12, respectively, containing 0.6% agarose (upper layer). The bottom layer of solidified matrix was made up of 1% agarose in 2X RPMI-1640 or 2X DMEM/F-12. Cells were plated in a 6-well plate with a concentration of 5 × 10^4^ cells for each well. All cells were maintained at 37 °C with 5% CO_2_ for 3 weeks. Colonies were stained with crystal violet and photographed by inverted microscope. ImageJ software was used to quantify the diameter of colonies, which were considered formed by more than two cells (diameter ≥25 μm). Instead, a diameter <25 μm is referred to cell debris, one, or two cells.

### 2.7. Cell Cycle Analysis

Cell cycle distribution of Panc1 parental cells and CSCs at 2, 4, and 8 weeks of culture was analyzed using propidium iodide (PI)-stained cells. Briefly, 3 × 10^5^ cells were washed with 1X PBS, incubated 5 min with 0.1% sodium citrate dihydrate, 0.1% Triton X-100, 200 μg/mL RNase A, 50 μg/mL propidium iodide (Roche Molecular Biochemicals, USA), lysed with a syringe needle, and analyzed using a flow cytometer (FACSCanto, Becton Dickinson, USA). The percentage of nuclei in the various stages of the cell cycle was determined using the ModFit LT software (v. 5.0.9; Verity Software House, Topsham, ME).

### 2.8. Senescence Analysis

Panc1 parental cells and CSCs at 2, 4, and 8 weeks of culture were plated in 24-well cell culture plates (3 × 10^4^ cells/well) in their respective medium and were incubated at 37 °C with 5% CO_2._ After 48 h, when the cells reached a confluence of 70%, the growth media was removed from the cells and the cells were washed with 1× PBS. The cells were fixed with Fixative Solution at room temperature for 30 min, washed with 1× PBS, and incubated with β-Galactosidase Staining Solution at 37 °C overnight in dry incubator. The cells were visualized and counted with inverted microscope (Axio Vert. A1, Zeiss, Germany): Blue color due to β-Galactosidase activity at pH 6 is present only in senescent cells and not in pre-senescent, quiescent or immortal cells. We used Senescence β-Galactosidase Staining Kit supplied by Cell Signaling Technology.

### 2.9. Omic Analyses

For proteomics analyses, the data indicated in this paper are extrapolated from an ongoing study on the proteomics and lipidomics analysis of CSCs. In particular, the reported proteome modulations were obtained by a label free approach, as previously described [22]. Briefly, total proteins from Panc1 parental cells and CSCs grown for 2, 4, and 8 weeks were extracted and analyzed by LC-MS/MS using the SWATH method and the differentially expressed proteins were selected according to a statistically significant *p*-value <0.05 and a fold change threshold of 1.5. The UNIPROT ID of the identified modulated proteins reported here are the following: Aldolase A (P04075-2), ATP-dependent citrate lyase (P53396-3), cytochrome c oxidase subunit 2 (P00403), enolase (P06733), glucose-6-phosphate isomerase (P06744-2), NADH dehydrogenase iron-sulfur protein 3 (O75489), phosphoenolpyruvate-carboxykinase (Q16822), phosphofructokinase (Q01813-2), pyruvate dehydrogenase E1 component subunit alpha (P08559), pyruvate dehydrogenase E1 component subunit beta (P11177), succinate dehydrogenase flavoprotein subunit A (P31040), succinate dehydrogenase iron-sulfur subunit B (P21912), and transketolase (P29401).

For metabolomics analyses, 1x10^6^ cells per group (Panc1 parental cells, CSCs and AdCSCs) were used. To lyse cells, cell culture pellets were re-suspended in 0.15 mL of ice-cold ultra-pure water (18MΩ). The tubes were plunged into dry ice (or a circulating bath at −25 °C) for 30 s and then into a water bath at 37 °C for 30 s. Six hundred µl of −20 °C methanol and then 450 µL of −20 °C chloroform were added to each tube. The contents of the tubes were mixed by gentle rotation every 5 min for 30 min. Subsequently, 0.15 mL of ice-cold pH-adjusted ultra-pure water was added to each tube and these were centrifuged at 1000× *g* for 1 min at 4 °C, before being transferred to −20 °C for 2–8 h. After thawing, liquid phases were recovered and an equivalent volume of acetonitrile was added to precipitate any residual protein. The tubes were then transferred to a refrigerator (4 °C) for 20 min, centrifuged at 10,000× *g* for 10 min at 4 °C and the collected supernatants were dried to obtain visible pellets. Finally, the dried samples were re-suspended in 0.1 mL of water, 5% formic acid and transferred to glass autosampler vials for LC/MS analysis. Twenty microliters of supernatants (three technical replicates) were injected into an Ultra High-Performance Liquid Chromatography (UHPLC) system (Ultimate 3000, Thermo Fisher, USA) and run in positive ion mode. Chromatographic separations were carried on a Reprosil C18 column (2.0 mm × 150 mm, 2.5 μm, Dr Maisch, Germany), at a column temperature of 30 °C and a flow rate of 0.2 mL/min. A 0–100% linear gradient of solvent A (ddH2O, 0.1% formic acid) to B (acetonitrile, 0.1% formic acid) was employed over 20 min, returning to 100% A in 2 min and a 6 min post-time solvent A hold. The UHPLC system was coupled online with a Q Exactive (Thermo Fisher, USA) mass spectrometer scanning in full MS mode (2 μscans) at 70,000 resolution in the 67 to 1000 m/z range, target of 1 × 10^6^ ions and a maximum ion injection time (IT) of 35 milliseconds. Source ionization parameters were: spray voltage, 3.8 kV; capillary temperature, 300 °C; sheath gas, 40; auxiliary gas, 25; S-Lens level, 45. Calibration was performed before each analysis against positive ion mode calibration mixes (Piercenet, Thermo Fisher, USA) to ensure sub ppm error of the intact mass. Raw files of replicates were exported and converted into mzXML format through MassMatrix, then processed by MAVEN software (http://maven.princeton.edu/). Mass spectrometry chromatograms were elaborated for peak alignment, matching and comparison of parent and fragment ions, and tentative metabolite identification (within a 2 ppm mass-deviation range between observed and expected results against the imported KEGG database). Statistical analyses were performed on the entire metabolomics data set by using the MetaboAnalyst 4.0 software (http://metpa.metabolomics.ca/). Before the analysis, raw data were normalized by sum and auto-scaled. False discovery rate (FDR) was used for controlling multiple testing. Pathway analysis was performed utilizing the MetPA (Metabolomic Pathway Analysis) web-based tool incorporated into MetaboAnalyst platform. Data for identified metabolites detected in all samples were submitted into MetPA with annotation based on common chemical names. Accepted metabolites were verified manually using HMDB, KEGG, and PubChem databases. The Homo sapiens pathway library was used for pathway analysis. Global test was the selected pathway enrichment analysis method, whereas the node importance measure for topological analysis was the relative betweenness centrality.

Controlled experiments of key metabolites, including glucose, glucose-6-phosphate, fructose 1,6-bisphosphate, lactate, fumarate, malate, and phosphogluconolactone, showed a similar regulation in Panc1 CSCs cultured in SsM with 1 or 2 g/L of glucose compared to Panc1 parental cells, indicating that the amount of glucose in the medium was not critical for the determination of cell metabolic features. Beside glucose, it is noteworthy that the two culture media (for details see “Cell lines” section) do not differ in l-glutamine concentration and that B27 does not contain either glucose or glutamine, indicating that the medium composition should not influence the metabolic features of the cells.

### 2.10. Oxygen Consumption Rate (OCR)

Mitochondrial oxygen consumption was assessed with the Seahorse Extracellular Flux Analyzer XF24 (Seahorse Bioscience, USA) as previously discussed [23]. The XF-24 cell culture plate was coated with 0.01% poly-l-lysine (P4832, Sigma-Aldrich, Germany) and incubated at room temperature for at least 1 h and half. Then, the plate was washed once with 1× PBS and dry at room temperature. Panc1 parental cells and CSC aggregates/spheres, with a size >40 μm, were plated in the treated plate (6 × 10^4^ cells/well and 1 × 10^5^ cells/well, respectively) in the presence of their respective medium and were incubated at 37 °C with 5% CO_2_ overnight. Assays were initiated by centrifuging the seahorse plate for 15 min at 5000× *g*, replacing the growth medium with 670 µL serum and antibiotic-free unbuffered DMEM (Ph 7.4) supplemented with 2 mM glutammate and 1 mM pyruvate, and by incubating the plate at 37 °C for 20 min. After the probes were calibrated, the OCR was detected in basal condition and in presence of the following compounds regulating mitochondrial respiration: oligomycin (ATP synthase inhibitor; 1 µM), carbonylcyanide p-trifluoro methoxyphenylhydrazone (FCCP) (uncoupler; 400 nM), rotenone (complex I inhibitor; 1 µM), and antimycin A (complex III inhibitor; 1 µM). The OCR values were normalized on protein amount. Briefly, after the measurements, cells were collected, lysed in extraction buffer containing 150 mM NaCl, 20 mM TRIS/HCL pH7.4, 5mM EDTA, 1% triton, and 10% glycerol and quantified by Bradford Assay.

### 2.11. Zebrafish

Zebrafish experiments were performed at the Interdepartmental Centre for Experimental Research (CIRSAL) of the University of Verona. Zebrafish embryos obtained from Nacre adults [24] were maintained under standard husbandry conditions and mated according to standard procedures. For xeno-transplantation experiments, zebrafish embryos were dechorionated at 2 days post fertilization (dpf), anesthetized with tricaine and positioned ventrally in 3% methylcellulose on a plastic mould. Panc1 parental cells and CSCs at 2, 4, and 8 weeks of culture were washed with 1× PBS and stained with Vybrant Cell-Labeling Solution for 10 min at 37 °C according to manufacturer instructions. Then, cells were washed with 1× PBS, resuspended in RPMI 1640 without serum and keep on ice until the injections. Stained cells were loaded in a glass capillary needle and microinjected into the yolk of the zebrafish embryos by means of a WPI PicoPump apparatus under a Leica M80 stereomicroscope. Injected embryos were grown at 33 °C and monitored from 1 day post injection (dpi) up to 4 dpi (experimental endpoint). Images were taken using a Leica MZ16F fluorescence microscope equipped with a DFC7000T camera. All the analyses on zebrafish larvae have been performed in at least two independent experiments and a chi-square test was used to determine significance. For each experimental condition at least 70 larvae have been examined and scored. All experiments were carried in accordance to Italian law on animal experimentation (D.L. 4 March 2014, n.26).

### 2.12. Statistical Analysis

ANOVA (post hoc Bonferroni) analysis by GraphPad Prism 5 (GraphPad Software, Inc., USA) or student’s *t*-test (two-tailed) were conducted. *p*-values < 0.05, 0.01, or 0.001 are considered significantly different.

## 3. Results

### 3.1. Long-Term Culture in the Stem-Specific Medium Elicits the Stem and Mesenchymal Properties in PDAC Cells

**Figure 1:**
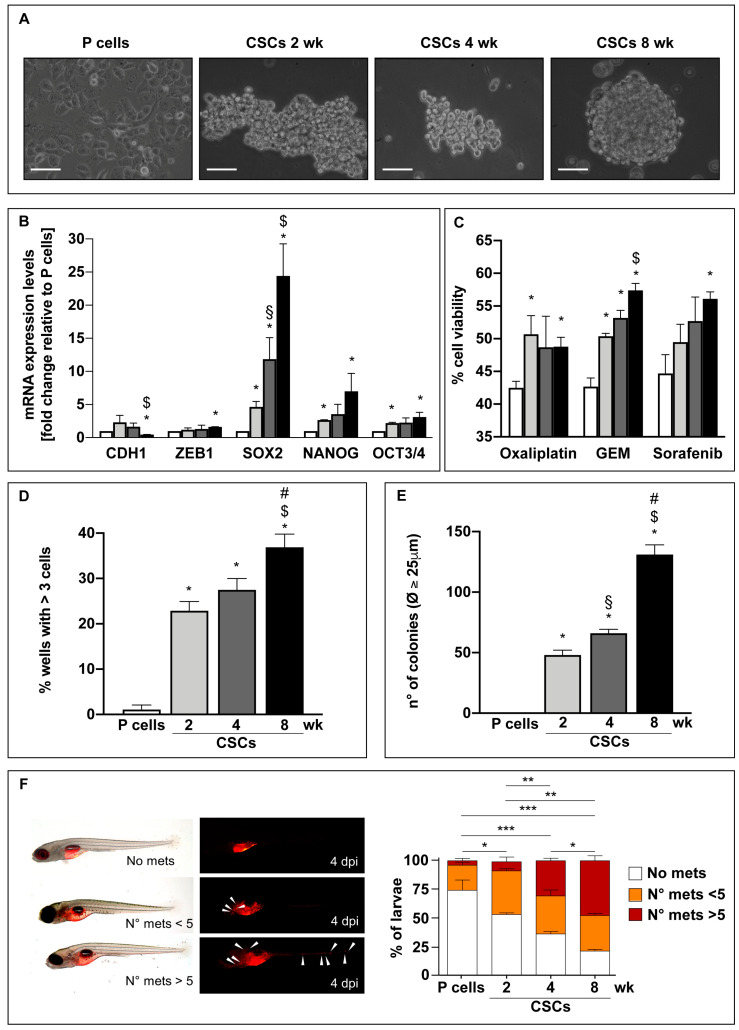
Panc1 cells cultured in the stem-specific medium for 2, 4, and 8 weeks enhance their epithelial-to-mesenchymal transition (EMT)/stem properties. (**A**) Bright field representative images of Panc1 parental cells (P) and Panc1 cancer stem cells (CSCs) cultured in the stem-specific medium for 2, 4, and 8 weeks. Scale bar: 100 μm. (**B**) qPCR analysis of the basal expression of EMT markers, i.e., CDH1 and ZEB1, and stem markers, i.e., SOX2, NANOG, and OCT3/4, in Panc1 P cells and CSCs. The values are reported as fold change relative to P cells. (**C**) Cell viability analysis of Panc1 P cells and CSCs treated with 100 μM Oxaliplatin or 50 μM Gemcitabine (GEM) or 20 μM Sorafenib for 48 h. (**D**) Clonogenic assay: The percentage of wells in 96 well-plates with more than 3 cells is reported after 15 days of the seeding of 1 cell/well of Panc1 P cells or CSCs. (**E**) Soft agar colony formation assay: The number of Panc1 P or CSC colonies with diameter ≥ 25 μm grown in soft agar is reported after 21 days of 5 × 10^4^ cells seeded in each well. Histograms legends: white: P cells; light gray: CSCs 2 weeks; dark grey: CSCs 4 weeks; black: CSCs 8 weeks. (**F**) Panc1 parental cells and CSCs were stained with Vybrant Cell Labeling Solution (red) and injected into the yolk of zebrafish larvae at 2 dpf. The percentage of larvae with metastasis-forming cells was counted at 4 days post injection (dpi) in each experimental condition and reported in the histograms. Histograms legends: No mets.= larvae with no tumor cells outside the yolk; n mets < 5 = larvae with <5 tumor cells outside the yolk; n mets > 5 = larvae with >5 tumor cells outside the yolk. Histograms legends: white: P cells; light gray: CSCs 2 weeks; dark grey: CSCs 4 weeks; black: CSCs 8 weeks. Values are the means (± SE) of at least three independent biological replicates for in vitro assays and two biological replicates for in vivo assay. Statistical legend: *p* < 0.05 (*), *p* < 0.01 (**), or *p* < 0.001 (***) P cells versus CSCs; (§) CSCs 2 weeks versus CSCs 4 weeks; ($) CSCs 2 weeks versus CSCs 8 weeks; (#) CSCs 4 weeks versus CSCs 8 weeks.

Several papers attempted to delineate CSC metabolism reporting contrasting results due to the different methodological approaches and to the generally short in vitro culture periods, leaving open the matter on which is the metabolic setting of CSCs. In this paper, we cultured PDAC cells in vitro in a stem-specific medium (SsM) (see materials and methods) at three different time points, short-term (2 weeks), medium-term (4 weeks), and long-term (8 weeks). This approach allowed us to study and follow during the time the phenotypic and metabolic changes of progressively de-differentiated CSCs, helping to shed light on the stemness acquisition by cancer cells. Noteworthily, our preliminary data show that when PDAC cells are cultured in the SsM, they quickly change their morphology by starting to grow in suspension approximately after 24 h (Supplementary Figure S1A). This observation suggests that our cellular model obtained through the use of the SsM preferentially derives from de-differentiation of the original parental cells rather than selection of pre-existing stem cells among the differentiated pull of parental cells. During the de-differentiation process, we observed that all the four analyzed PDAC cell lines change their morphology (Figure 1A, for Panc1 cells and Supplementary Figure S1B, for PaCa44, PaCa3, and PC1J cells). Indeed, Figure 1A shows that at 2 weeks, Panc1 cells form random floating aggregates, which decrease in volume at 4 weeks by finally forming big and round-shaped spheres at 8 weeks of culture, maintaining these features even after 12 weeks of culture (Supplementary Figure S1C). Together with the acquisition of a spherical morphology, long-term culture induces more mesenchymal and stem features in comparison to the short- and medium-term culture. In particular, only after 8 weeks of culture, Panc1 cells show a significantly decreased expression level of the epithelial marker E-cadherin gene (*CDH1*), together with an increased expression of the mesenchymal marker *ZEB1*, strongly suggesting the loss of the epithelial state and the acquisition of the mesenchymal phenotype (Figure 1B), in line with the formation of tumorspheres (Figure 1A). In parallel with these results, three known stem markers are highly expressed in long-term culture. Indeed, SOX2 mRNA expression is significantly higher after 8 weeks of culture in comparison to parental cells and to shorter culture periods. Also NANOG and OCT3/4 mRNA expression levels are significantly increased in long-term culture in comparison to parental cells (Figure 1B). Concerning the other analyzed PDAC cell lines, they all show a significant increase or a trend of increase of the three stem markers SOX2, NANOG, and OCT3/4 after 4 and 8 weeks of culture in comparison to parental cells (Supplementary Table S1).

Since it has been widely reported that CSCs are strongly chemoresistant thus favoring malignant progression, metastasis, and cancer recurrence [10], we analyzed the in vitro effects of three different chemotherapeutic agents on the viability of Panc1 cells. Panc1 cells cultured in SsM were generally more resistant to Oxaliplatin, Gemcitabine (GEM) or Sorafenib compared to parental cells and the 8-week culture was the most resistant (Figure 1C).

To further investigate the acquisition of the stem features by cells cultured in SsM, we performed clonogenic assays to evaluate the capability of single cells to undergo clonal expansion, a sensitive indicator of the undifferentiated state of the cell [25]. We found that Panc1 cells cultured in SsM possess high clonogenic capability at all the three time points, whereas parental cells do not have this ability, indicating a more differentiated phenotype (Figure 1D). The diameter of the spheres formed by cells that were able to give rise to clones (Figure 1D) have been measured 30 days after the plating (Supplementary Figure S2A,B). Furthermore, it is noteworthy that single cells derived from 8-week CSCs show the highest clonogenic capability, further supporting a heightened acquisition of stem features during long-term culture (Figure 1D). Another hallmark of CSCs is represented by their capability to grow independently of a solid surface. To assess this property on the SsM cultured cells, we performed the soft agar colony assay, a well-established in vitro method that permits to analyze anchorage-independent growth based on the colony formation capability of the cells cultured on the agarose matrix [26]. Cells cultured in SsM increased their colony formation capability with the highest extent in cells originated from 8-week CSCs (Figure 1E and Supplementary Figure S2C), further confirming that long-term culture confers stem properties.

Finally, in order to definitively ascertain the acquisition of stem features by the cells, their behavior in vivo was assayed. In our previous article, we have shown that PDAC CSCs cultured in SsM for about two weeks possess higher tumor-seeding ability in mice in comparison to parental cells [6]. Since it is known that cells harboring stem and mesenchymal features possess a higher migratory ability [27], here we aimed to evaluate the metastatic capacity of the obtained cells by injecting Panc1 cells previously cultured at the three time points in SsM and the relative parental cells in zebrafish (*Danio rerio)*, an animal model that has recently contributed to major insights in the development and progression of PDAC [28,29]. Indeed, Teng et al. previously reported that different tumor cell types, including pancreatic cancer cells, injected in zebrafish larvae show a degree of cell metastasis in fish that is proportional to their invasion potential in vitro [30]. We injected tumor cells into 48 h post fertilization (hpf) transparent embryos and evaluated the spread of cells 4 days later, a period which allows cancer cells to survive and metastatize since zebrafish larvae have not yet developed an adaptive immune system [30]. The data obtained show that, in the 74% of the larvae, the injected parental cells were confined in the site of injection (yolk), while in 24% of the sample a migration of tumor cells was observed (Figure 1F). Conversely, cells previously cultured in vitro in the SsM show a progressive ability to migrate and give rise to metastasis in an increasing number of larvae (Figure 1F). The highest number of metastasis-forming cells (79%) was observed in the larvae injected with the cells previously cultured in SsM for 8 weeks indicating that a direct correlation between the spreading ability of the cells and the SsM incubation time can be assumed. These in vivo data additionally confirm that cells cultured in SsM for long periods further increase their aggressiveness and metastatic potential, which are known features of tumor cells with stem properties [31], and that these characteristics are maintained also after their injection in vivo, represented here by Zebrafish. 

Altogether, these data indicate that cells cultured in SsM at all the three time points possess stem features. Therefore, we have considered all of them as “cancer stem cells” (CSCs), even though long-term culture cells show the highest grade of stemness.

### 3.2. Long-Term Cultured CSCs Enter a Quiescent State

**Figure 2:**
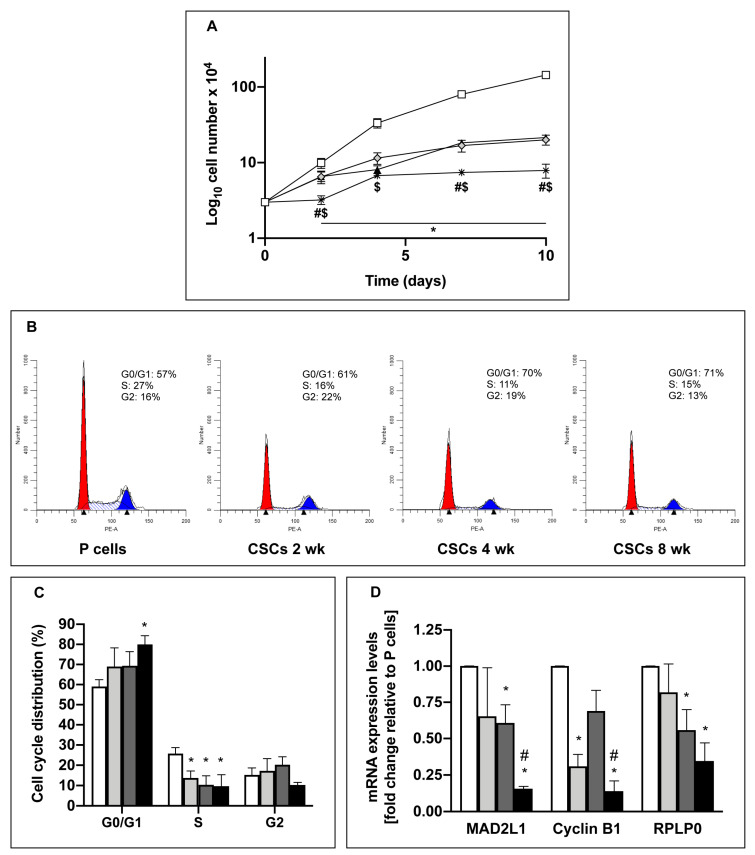
Long-term culture of cancer stem cells (CSCs) favors the entrance in a quiescent state. (**A**) Proliferation rate of Panc1 P and CSCs cultured in their specific-medium for 2, 4, 7, and 10 days. 5 × 10^3^ cells/well have been plated for each condition at time 0. Legend: White squared: P cells; gray rhombus: CSCs 2 weeks; black triangle: CSCs 4 weeks; star: CSCs 8 weeks. (**B**) Representative histograms of cell cycle analysis for each condition. (**C**) Cell cycle distribution of Panc1 P cells and CSCs through flow cytometry. (**D**) qPCR analysis of the quiescent markers MAD2L1, Cyclin B1, and RPLP0 in Panc1 P cells and CSCs. The values are reported as fold change relative to P cells. Histograms legends: white: P cells; light gray: CSCs 2 weeks; dark grey: CSCs 4 weeks; black: CSCs 8 weeks. Values are the means (± SE) of at least three independent biological replicates. Statistical legend: *p* < 0.05 (*) P cells versus CSCs; ($) CSCs 2 weeks versus CSCs 8 weeks; (#) CSCs 4 weeks versus CSCs 8 weeks.

We and others have previously shown that CSCs proliferate slower than differentiated cells [6,32]. Here, we find that cells cultured in SsM lose their proliferative capacity over time, with long-term cultured cells having the lowest proliferation rate (Figure 2A). Indeed, 8-week CSCs show a 3.6- or 2-fold higher doubling time than parental cells or 2–4-week CSCs, respectively (Table 1, first column), indicating their entrance in a slow-cycling state. In accordance, the cell cycle distribution analysis shows that all CSCs have a significant S phase drop in comparison to parental cells and only 8-week CSCs had a significant accumulation in the G0/G1 phase (Figure 2B and C). In line with our data, other authors showed that quiescent cells have a doubling time that is about 4 fold higher than the corresponding proliferating cells [33]. However, we aimed to clarify whether the slow-cycling properties of CSCs are due to quiescence or senescence. Generally, quiescence is a reversible dormant-state [34], whereas senescence is a permanent state of cell cycle arrest that takes place due to aging and represents a degenerative process [35]. In order to exclude senescence, we performed the beta-galactosidase senescent assay that shows, in all conditions, only a minimal amount of senescent cells (less than 3%) (Supplementary Figure S2D). Hence, to demonstrate that low proliferation rate and G0/G1 phase accumulation of 8-week CSCs is due to quiescence, we analyzed the expression levels of three proteins previously shown to be decreased in quiescent cells: The mitotic spindle assembly checkpoint protein MAD2L1, the cell cycle regulator cyclin B1 [36,37], and a ribonucleoprotein (RPLP0), that belongs to a family involved in translation [38,39]. Figure 2D shows that: (i) 8-week CSCs had significantly lower expression levels of MAD2L1 and cyclin B1 in comparison to parental cells and to 4-week CSCs, and (ii) RPLP0 had a progressively decreased trend of expression during de-differentiation. Also medium- and long-term cultured CSCs derived from PaCa44, PaCa3, and PC1J cells showed a significant decrease or a trend of decrease of MAD2L1, cyclin B1 and RPLP0 expression, further supporting the observation that stem induction by SsM culture is cell line-independent (Supplementary Table S1). Altogether, these data indicate that the induction of the mesenchymal/stem state of long-term cultures in SsM affects cell proliferation, cell cycle distribution, and favors the entrance in a quiescence state, which is considered another typical stem marker [40].

### 3.3. CSCs Cultured in the Differentiated-Cell Medium Re-Acquire Epithelial Features and Exit from the Quiescent State

**Figure 3:**
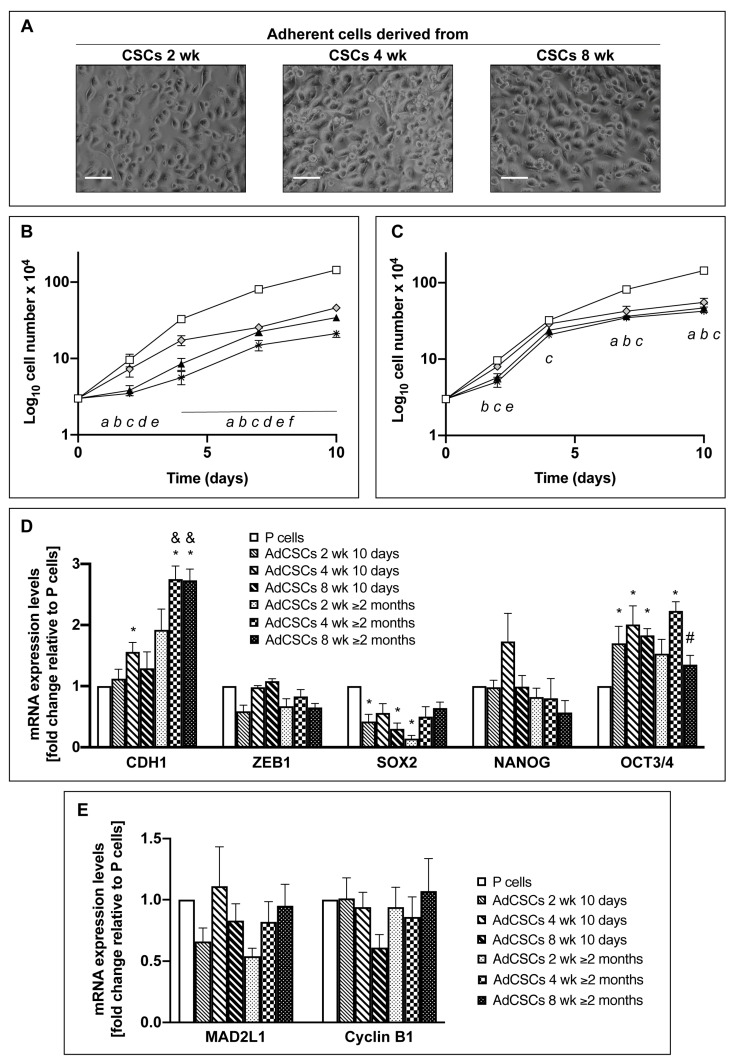
CSCs cultured in the differentiated-cell medium re-acquire epithelial features and exit from the quiescent state. (**A**) Bright field images of adherent cells derived from Panc1 CSCs after 24 h of culture in the differentiated-cell medium (DM). Scale bar: 100 μm. Proliferation rate of Panc1 P and adherent cells derived from Panc1 CSCs after 10 days (**B**) and 2 months (**C**) of culture in DM. 5 × 10^3^ cells/well have been plated for each condition at time 0. Legend: White squared: P cells; gray rhombus: adherent cells derived from CSCs 2 weeks; black triangle: adherent cells derived from CSCs 4 weeks; star: adherent cells derived from CSCs 8 weeks. Statistical legend: *p* < 0.05 (*a*) P cells versus AdCSCs 2 weeks; (*b*) P cells versus AdCSCs 4 weeks; (*c*) P cells versus AdCSCs 8 weeks; (*d*) AdCSCs 2 weeks versus AdCSCs 4 weeks; (*e*) AdCSCs 2 weeks versus AdCSCs 8 weeks; (*f*) AdCSCs 4 weeks versus AdCSCs 8 weeks. (**D**) qPCR analysis of the basal expression of EMT markers, i.e., CDH1 and ZEB1, and stem markers, i.e., SOX2, NANOG, and OCT3/4, in Panc1 P cells and adherent cells derived from Panc1 CSCs after 10 days and 2 months of culture in DM. The values are reported as fold change relative to P cells. (**E**) qPCR analysis of the quiescent markers MAD2L1 and Cyclin B1 in Panc1 P cells and adherent cells derived from Panc1 CSCs after 10 days and 2 months of culture in DM. The values are reported as fold change relative to P cells. Legend: parental cells: AdCSCs: adherent cells derived from the indicated CSCs cultured in DM for 10 days or more than 2 months. Values are the means (± SE) of at least three independent biological replicates. Statistical legend: *p* < 0.05 (*) P cells versus AdCSCs; (#) AdCSCs 4 weeks versus AdCSCs 8 weeks; (&) AdCSCs cultured in DM for 10 days versus the same cells cultured in DM for more than 2 months.

Since quiescence is a reversible phase from which cells may re-enter the cell cycle in response to physiological cell stimuli, we re-cultured CSCs in the differentiated-cell medium (DM) (see materials and methods). Figure 3A shows that, after 24 h of culture in DM, cells drastically changed their morphology by forming a monolayer adherent to the plate and decreased the expression level of SOX2 mRNA (data not shown). Furthermore, prolonged culture in DM for 10 days (Figure 3B) or for more than 2 months (Figure 3C) stimulates cells to accelerate their proliferation rate in comparison to their previous stem status, as shown by the decreased doubling time (Table 1 s and third columns). In particular, after 10 days of culture in DM, adherent cells derived from 2- and 4-week CSCs have a doubling time of less than 3 days and they maintain this doubling time even after more than 2 months of culture in DM. In contrast, adherent cells derived from 8-week CSCs take a longer time, more than 2 months, to reach the same doubling time than the other cells (Table 1), in line with the prominent quiescent features of the CSCs of origin. Furthermore, in comparison to their previous stem state, adherent cells derived from 8-week CSCs and cultured in DM for more than 2 months progressively lost their mesenchymal features, as shown by the increased expression of the E-cadherin gene and by the decreased expression of *ZEB-1*, and show a decreased expression of the stem markers SOX2, NANOG and OCT3/4 (Figure 3D and Table 2). Finally, the adherent cells derived by all the CSCs express levels of the quiescent markers similar to those of parental cells (Figure 3E and Table 2). A further confirmation of the gene expression pattern differences among the stem cells and the derived-adherent cells is appreciable also in the Heatmap representation of the unsupervised hierarchical clustering of target gene expression across all samples (Supplementary Figure S3). Altogether, these data further support the strongest stem features of the long-term cultured CSCs, which need longer time to undergo the mesenchymal-to-epithelial transition (MET) in comparison to short- and medium-term cultured CSCs.

### 3.4. CSCs Modify Their Metabolic Settings Towards the Acquisition of Quiescence Properties

In order to determine whether CSC plasticity, which causes cells to progressively increase their stem and mesenchymal features finally gaining the quiescent state, is associated with metabolic changes, we performed an integrative analysis of Panc1 cell and CSC metabolic features by using qPCR, metabolomics, proteomics, and Seahorse technology. At a first glance, MetaboAnalyst software analysis of the metabolomics data highlights that CSCs possess stage-specific metabolic phenotypes during the progressive de-differentiated stages (Supplementary Figure S4, for details see figure legend) and identifies several pathways as being significantly impacted by the transition to quiescence. We focused on the main energy metabolism-related pathways, including glycolysis, pentose phosphate pathway, tricarboxylic acid cycle, electron transport chain, and oxygen consumption. Below, we describe the data obtained with the different techniques separated for the different time points.

### 3.5. CSCs Demonstrate an Upregulation of Glycolysis and the PPP, and Downregulation of Oxidative Phosphorylation, at 2 Weeks of Culture

**Figure 4:**
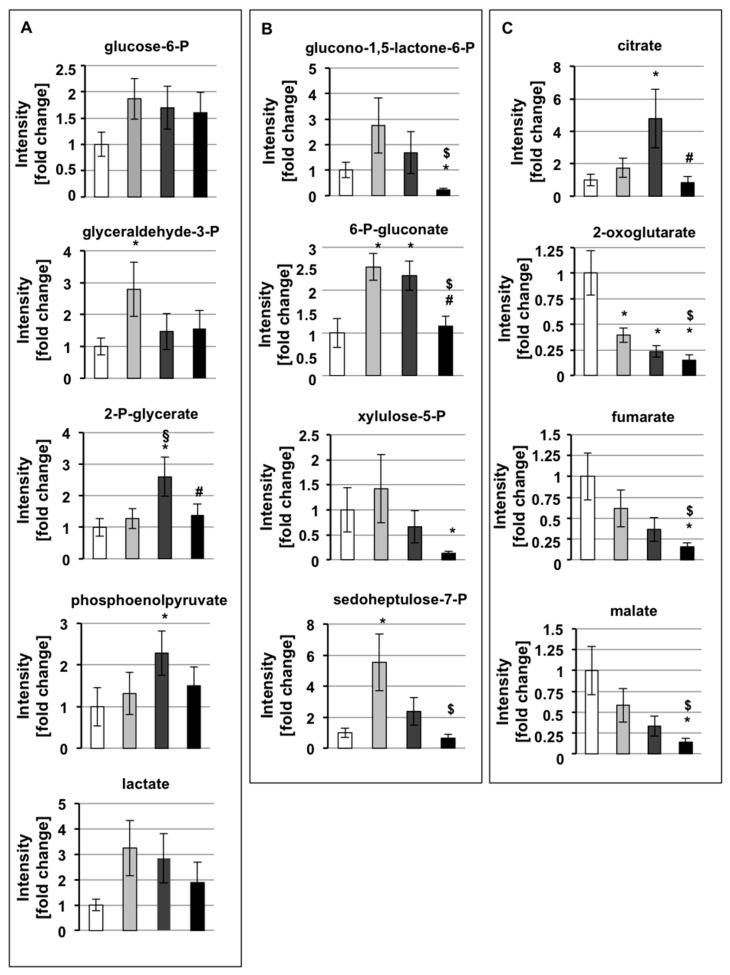
Progressively CSC de-differentiation is accompanied by metabolic changes. Metabolomic quantification of intracellular levels of metabolites reported as fold change relative to parental cells. Metabolites from glycolysis (**A**), pentose phosphate pathway (**B**), and tricarboxylic acid cycle (**C**) in Panc1 P cells and CSCs cultured for 2, 4, and 8 weeks. Legend: white: parental (P) cells; light gray: CSCs 2 weeks; dark grey: CSCs 4 weeks; black: CSCs 8 weeks. Values are the means (± SE) of three technical replicates and three independent biological replicates. Statistical legend: *p* <0.05 (*) P cells versus CSCs; (§) CSCs 2 weeks versus CSCs 4 weeks; ($) CSCs 2 weeks versus CSCs 8 weeks; (#) CSCs 4 weeks versus CSCs 8 weeks.

**Figure 5:**
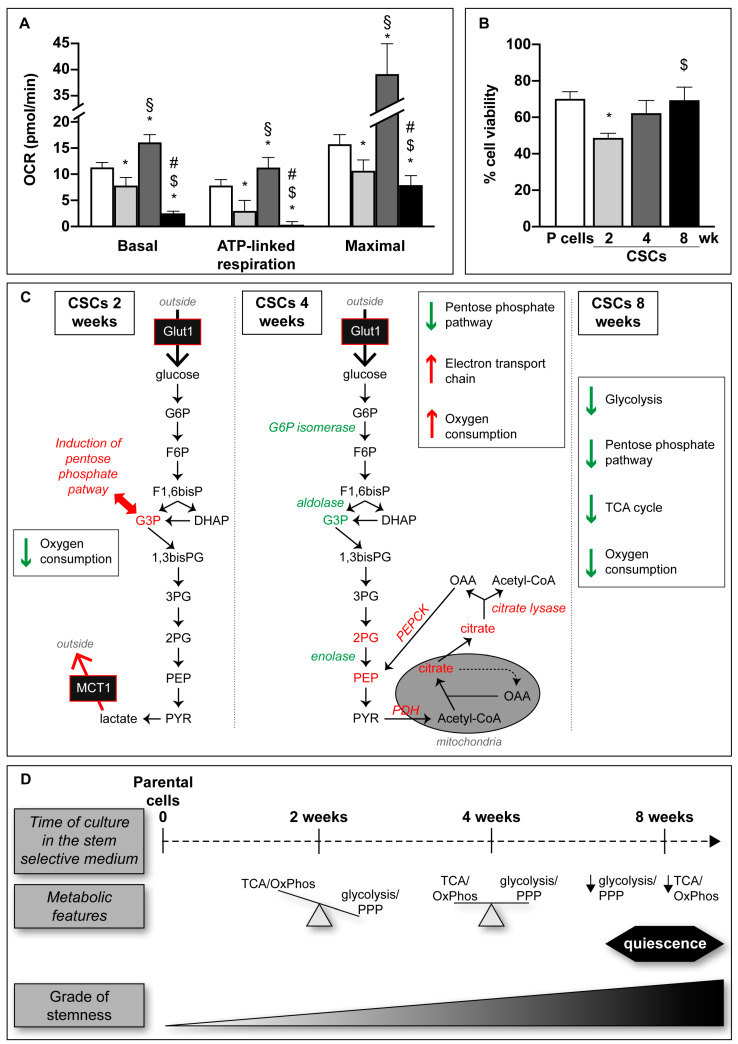
Metabolic features of CSCs. (**A**) Seahorse analysis of Panc1 P and CSCs cultured in their specific-medium; 6 × 10^4^ cells/well and 1 × 10^5^ cells/well of P and CSCs respectively have been plated for each condition. After 24 h, cells were treated with FCCP (400 nM), oligomycin (1 µg), and a mix of antimycin and rotenone (both 1 µM) and then analyzed. Histograms legends: white: parental (P) cells; light gray: CSCs 2 weeks; dark grey: CSCs 4 weeks; black: CSCs 8 weeks. (**B**) Cell viability analysis of Panc1 P cells and CSCs treated with 50 nM AZD3965, the MCT-1 inhibitor, for 72 h. Molecular (**C**) and schematic (**D**) representations of the data presented in this paper showing the metabolic plasticity of CSCs. In green are indicated the metabolites or metabolic enzymes whose intracellular level or expression, respectively, is decreased. Instead, are reported in red when their level is increased. In italic are reported the enzyme’s name. Values are the means (± SE) of three independent biological replicates. Statistical legend: *p* <0.05 (*) P cells versus CSCs; (§) CSCs 2 weeks versus CSCs 4 weeks; ($) CSCs 2 weeks versus CSCs 8 weeks; (#) CSCs 4 weeks versus CSCs 8 weeks. Legends: glucose transporter (Glut1); glucose-6-phosphate (G6P); fructose-6-phosphate (F6P); fosfofructokinase (PFK); fructose 1,6-bisphosphate (F1,6bisP); glyceraldehyde-3-phosphate (G3P); dihydroxyacetone phosphate (DAHP); 1,3-bisphospho glycerate (1,3bisPG); 3-phosphoglycerate (3PG); 2-phosphoglycerate (2PG); phosphoenolpyruvate (PEP); pyruvate (PYR); monocarboxylate transporter-1 (MCT1); pyruvate dehydrogenase (PDH); oxaloacetate (OAA); phosphoenolpyruvate carboxykinase (PEPCK); tricarboxylic acid (TCA) cycle; oxidative phosphorylation (OxPhos); pentose phosphate pathway (PPP).

While we found that the mRNA expression of the glucose transporter Glut1 (Supplementary Figure S5) and the glycolytic intermediate glucose-6-phosphate (Figure 4A) was increased similarly in all CSCs in comparison to parental cells, 2-week CSCs demonstrated an increase in the glycolytic intermediate glyceraldehyde-3-phosphate compared to other time points (Figure 4A). Two-week CSCs have the highest trend to increase the pentose phosphate pathway (PPP) intermediates glucono-1,5-lactone-6-phosphate, xylulose-5-phosphate, and sedoheptulose-7-phosphate in comparison to parental cells, 4- and 8-week CSCs (Figure 4B). In accordance with the previously described higher level of glyceraldehyde-3-phosphate in 2-week CSCs (Figure 4A), the expression of the enzyme transketolase, which drives the formation of glyceraldehyde-3-phosphate and sedoheptulose-7-phosphate in PPP, is 2.5-fold higher in short-term cultured CSCs, compared to parental cells. Importantly, oxidative phosphorylation is downregulated (Figure 5A), particularly ATP-linked respiration, suggesting a higher reliance on glycolytic metabolism to supply cellular ATP. Altogether these data indicate that, at the beginning of the de-differentiation process, CSCs show increased metabolites in the PPP and glycolysis pathway, and downregulation of oxidative phosphorylation.

### 3.6. CSCs Demonstrate an Upregulation of Oxidative Phosphorylation at 4 Weeks of Culture

We found that keeping CSCs in culture for two more weeks (4-week CSCs) led to the accumulation of the down-stream glycolytic intermediates 2-phosphoglycerate and phosphoenolpyruvate (Figure 4A). In contrast, almost all the metabolites of PPP, with the exception of 6-phospho-gluconate, show a decreased level in 4-week CSCs compared to 2-week CSCs, suggesting a reduction of PPP during the progression of cell de-differentiation. The parallel proteomic analyses highlight that, in comparison to 2-week CSCs, 4-weeks CSCs express significant lower levels of glucose-6-phosphate isomerase (0.35 fold), aldolase A (0.57 fold), and enolase (0.62 fold), with no change in the mRNA expression of enolase (Supplementary Figure 5), suggesting a post-translational regulatory mechanism. Phosphoenolpyruvate (PEP) accumulation may instead be induced by PEP-carboxykinase, which converts oxaloacetate into PEP. PEP-carboxykinase protein levels were 5.7- and 2.7-fold higher than parental cells and 2-week CSCs, respectively. Proteomics analysis also revealed that 4-week CSCs have 1.9-fold higher levels of both alpha and beta subunits of the PDH component E1 (PDH-E1), the enzyme that irreversibly converting pyruvate into acetyl-CoA to support a preferred entrance of acetyl-CoA into the TCA cycle in medium-term cultured CSCs. 4-week CSCs also had high intracellular levels of the TCA cycle metabolite citrate (Figure 4C), and proteomic data showing that the expression levels of the enzyme ATP-dependent citrate lyase is 5.2- and, in particular, 8-fold higher in 2- and 4-week CSCs, respectively, in comparison to parental cells. Citrate lyase catalyzes the cytosolic conversion of citrate into acetyl-CoA, which can be used by the cells for fatty acid synthesis or histone acetylation [41,42], and oxaloacetate, which can be converted into PEP by PEP-carboxykinase, shown above to possess the highest expression level in 4-week CSCs. Other TCA cycle intermediates, such as 2-oxoglutarate, fumarate, and malate, were decreased in all CSCs in comparison to parental cells (Figure 4C).

Interestingly, proteomic analysis revealed that NADH dehydrogenase (complex I), succinate dehydrogenase (complex II), and cytochrome C oxidase (complex IV) were highly expressed in 4-week CSCs in comparison to parental cells and to CSCs cultured for shorter and longer periods. Indeed, in 4-week CSCs, NADH dehydrogenase is 3 and 2 fold higher than in parental cells and 8-week CSCs, respectively; succinate dehydrogenase subunit A (SDHA) is 7.3, 2.2, and 3.5 fold higher than in parental cells, 2- and 8-week CSCs, respectively; succinate dehydrogenase subunit B (SDHB) is 4.3, 1.9, and 2.4 fold higher than in parental cells, 2- and 8-week CSCs, respectively; and cytochrome C oxidase is 1.9 and 1.7 fold higher than parental cells and 2-week CSCs, respectively. Consistently, 4-week CSCs show the highest oxygen consumption rate (OCR) in Seahorse assay, particularly cellular respiration coupled with ATP production, suggesting reliance on oxidative phosphorylation (Figure 5A).

### 3.7. Week-Quiescent CSCs Downregulate Metabolic Processes

Finally, according to the higher doubling time and stem/mesenchymal features, CSCs cultured for longer periods (8-week CSCs) show decreased trends of the metabolites 2-phosphoglycerate, phosphoenolpyruvate, and lactate (Figure 4A) and enolase mRNA (Supplementary Figure S5) in comparison to the previous de-differentiation step (4 weeks of culture). In addition, all analyzed PPP metabolites were significantly decreased in 8-week CSCs, suggesting a slowdown of this pathway in quiescent cells. Furthermore, 8-weeks CSCs express significant lower levels of both PFK and PEP-carboxykinase protein (0.6- and 0.5-fold, respectively) compared to 4-week CSCs. Finally, 8-week CSCs had the lowest OCR of any time point, with ATP-linked respiration practically undetectable (Figure 5A). Together, these data indicate that long-term cultured CSCs diminish their metabolic requirements together with the deceleration of their proliferation.

Altogether these data demonstrate that, during progressive de-differentiation, CSCs shift from a more glycolytic to a more oxidative metabolism, accomplishing at 8 weeks of culture a global reduced metabolism together with the acquisition of a quiescent status. All the described metabolic changes in CSCs cultured in SsM at the different time points are schematically represented in Figure 5C.

### 3.8. Metabolic Phenotypes Confer Targetable

Because short-term cultured CSCs had higher glycolytic activity compared to other time points, we tested whether they would be sensitive to pharmacological inhibition of glycolytic flux. We used AZD3965, a specific inhibitor of the lactate transporter MCT-1 that is in phase I clinical trial [43]. Inhibition of MCT-1 impairs both the export and the import of lactate, causing pH alteration [44] and enhances the cytosolic levels of Ca^2+^ leading to apoptosis [45]. Short-term cultured 2-week CSCs showed the highest sensitivity to the AZD3965 (Figure 5B). These findings suggest that while both 2-week CSCs and 4-week CSCs have alterations of specific glycolytic intermediates, 4-week CSCs are less reliant on glycolysis due to upregulation of oxidative phosphorylation. Importantly, metabolic inhibitors are likely to target CSCs differently depending on their metabolic phenotypes.

### 3.9. Adherent Cells Derived from CSCs Escape the Quiescent State and Become Metabolically Active

**Figure 6:**
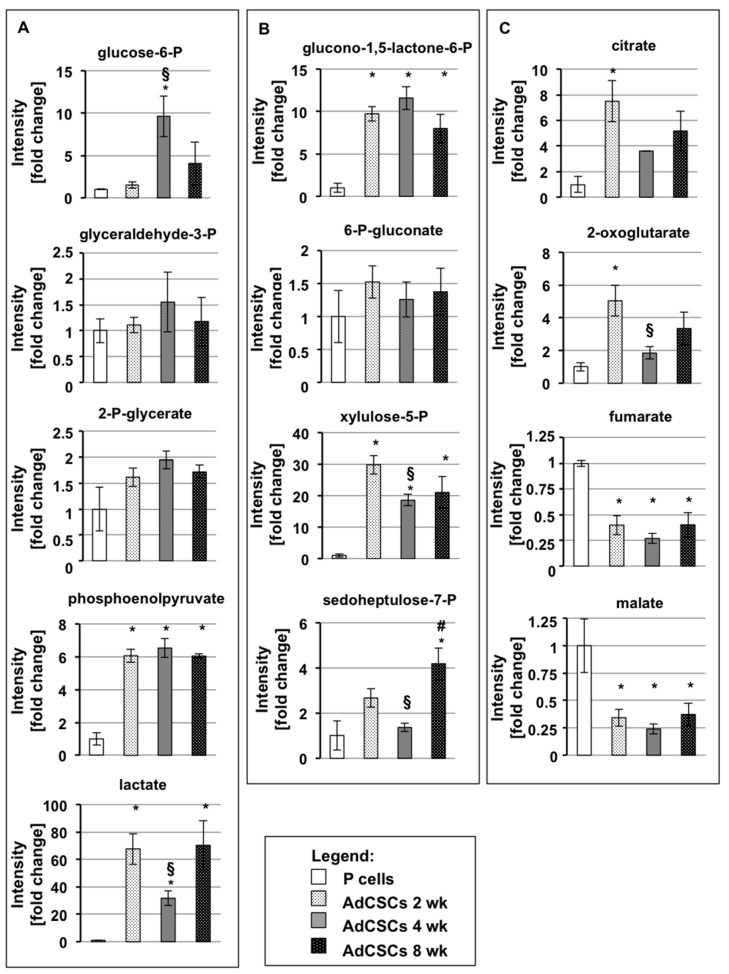
Metabolic features of adherent cells derived by CSCs. Metabolomic quantification of intracellular levels of metabolites reported as fold change relative to parental cells. Metabolites from glycolysis (**A**), pentose phosphate pathway (**B**), and tricarboxylic acid cycle (**C**) in Panc1 P cells and adherent cells derived from Panc1 CSCs after 2 months of culture in DM. Legend: P cells: parental cells; AdCSCs: adherent cells derived from the indicated CSCs cultured in DM for more than 2 months. Values are the means (± SE) of three technical replicates and two independent biological replicates. Statistical legend: *p* < 0.05 (*) P cells versus AdCSCs; (§) AdCSCs 2 weeks versus AdCSCs 4 weeks; (#) AdCSCs 4 weeks versus AdCSCs 8 weeks.

Finally, we analyzed the metabolic settings of adherent cells derived by CSCs that, as described above, re-acquire epithelial characteristics. The results of these analyses show that adherent cells derived from all the CSCs cultured for more than 2 months in DM undergo evident changes in glycolysis and PPP in comparison to their previous stem state (Figure 6A and B), especially regarding phosphoenolpyruvate, lactate, glucono-1,5-lactone-6-phosphate, and xylulose-5-phosphate. Furthermore, almost all the glycolytic metabolic intermediates, including glyceraldehyde-3-phosphate, 2-phospho-glycerate, and phosphoenolpyruvate, and PPP intermediates, including glucono-1,5-lactone-6-phosphate, 6-phospho-gluconate, and xylulose-5-phosphate, show very similar levels among all the adherent cells derived by CSCs (Figure 6A and B). Concerning TCA intermediates, in comparison to the CSCs of origin (Figure 4C), the levels of fumarate and malate remain unaltered, whereas the levels of citrate and, especially, of 2-oxoglutarate drastically change (Figure 6C). Altogether these data suggest that, when CSCs at the three de-differentiation steps are driven to re-differentiate, they acquire similar metabolic properties, also in accordance with their comparable proliferation rate reported in Table 1 (third column), by restoring similar metabolic parameters. In comparison to parental cells, two late glycolytic intermediates, i.e., phosphoenolpyruvate and lactate, and two PPP intermediates, i.e., glucono-1,5-lactone-6-phosphate and xylulose-6-phosphate, are strongly accumulated in all adherent cells derived from CSCs, suggesting a preferential commitment of these cells on glycolysis and PPP. Interestingly, the average of lactate accumulation for all the adherent cells derived from CSCs in comparison to parental cells is about 50 fold, suggesting a stronger aggressive feature of these re-differentiated cells. All these findings indicate that re-differentiated CSCs re-acquire epithelial morphology and proliferative properties similar to those of parental cells, however still maintaining some substantial differences that support their stronger malignancy.

## 4. Discussion

Pancreatic ductal adenocarcinoma (PDAC) cancer stem cells (CSCs) are a small subpopulation of cells within the tumor, representing about 0.1–1% of the total mass [31], whose role in tumorigenesis, metastasis formations, and relapse has been widely demonstrated [46]. Thus, to potentiate the effect of present therapies, the investigation of CSC features is crucial to identify specific targets. Up to now, many attempts have been made to characterize the metabolic status of CSCs with contrasting results. Here, we show for the first time that prolonged culture of PDAC cell lines in a stem-specific medium progressively lead to the enhancement of cell mesenchymal/stem properties associated with CSC metabolic plasticity, during which cells switch from a glycolytic to an oxidative metabolism to finally gain a quiescent state with a global metabolic shut-down. While proliferating cells devote much of their metabolic capacity to biosynthesis in order to synthesize the material necessary to form a new cell, quiescent cells are relieved of important metabolic requirements [47]. Consistently, we find that PDAC cells first shift their metabolic arrangement to a glycolytic metabolism while acquiring CSC properties (Figure 5D), which may help them to continue to proliferate in the new growth conditions. This is reflected also by the enhancement of pentose phosphate pathway, necessary for nucleotide and lipid syntheses, and by the high lactate production, required for the re-oxidation of NADH, which is indispensable for glycolysis to proceed. Then, during progressive de-differentiation in the same growth conditions, cells increase their oxidative metabolism by favoring pyruvate entrance into the TCA cycle and by raising the levels of intracellular citrate and citrate lyase expression, suggesting a tight epigenetic regulation [41], which could explain such quick transitions and metabolic changes. Furthermore, the implementation of the oxidative metabolism is also confirmed by the increased oxygen consumption rate coupled with ATP production. Finally, when the acquisition of stem features is consolidated, CSCs slowdown their global energy metabolism by entering in a slow-cycling/quiescence state. This situation is likely to occur in in vivo stem niches where a sub-population of tumor cells has the capacity to enter a non-dividing state in response, for instance, to lack of nutrients from blood supply, detachment from the substratum, or hypoxia [48]. The quiescent nature of CSCs represents an inherent mechanism that may partially explain chemotherapy resistance and recurrence in post-therapy cancer patients. In fact, during dormancy, cancer cells become clinically undetectable and resistant to cytotoxic drugs, and their reemergence from dormancy may determine higher aggressiveness [47]. This dormant state is likely to pose a challenge for the metabolic targeting of CSCs, as we find that they have low metabolic requirements, very low oxidative metabolism, and are concomitantly resistant to MCT1 inhibition. 

Accumulating evidence indicates that conventional therapies often fail to eradicate carcinoma cells that have entered the stem-state via activation of the epithelial-to-mesenchymal (EMT) program with the consequent tumor relapse [9]. Since the mechanism through which EMT determines the entrance into the stem state is still elusive [9], deep investigations of CSC induction through EMT are crucial for the generation of new effective therapies that specifically target these cells. The ability of cells that have exited the quiescent state to colonize secondary organs to form metastases and to generate relapse is closely related to the mesenchymal-to-epithelial transition (MET) [9]. Indeed, our data show that PDAC cells undergo EMT during CSC induction, and CSCs are able to undergo MET, re-differentiate, increase their proliferation by exiting from quiescence, and become metabolically active. These cells show a marked increase of lactate production in comparison to CSCs of origin and, especially, to parental cells. Consistently, in vivo findings show that lactate accumulation within the tumor correlates with poor clinical outcomes [49].

## 5. Conclusions

Since the eradication of CSCs remains a major challenge, our data have relevant implications in cancer therapy. Indeed, the therapeutic targeting of specific metabolic CSC features will help to avoid the pursuit of the quiescent state, which may represent the major culprit for cancer aggressiveness. Furthermore, our data indicate that it is necessary to take into account that the steps of tumor and metastasis development pass through a metabolic plasticity of cancer cells that has to be further characterized and validated in patients with different tumor types in order to develop the best strategy for an effective cancer eradication. To accomplish this aim, a major attention will be given to the identification of proteins that play a key role in the determination of EMT/MET and metabolic changes in CSCs, in order to determine a possible causal link between these two events.

## Figures and Tables

**Table 1:** Analysis of doubling time reported as day.

-	Doubling Time (Day)		
**-**	Standard culture conditions	Adherent cells derived from CSCs cultured in DM for 10 days	Adherent cells derived from CSCs cultured in DM for ≥ 2 months
**P cells**	2 ± 0.25	/	/
**CSCs 2 wk**	3.6 ± 0.05 ^*^	2.9 ± 0.31 ^*,£^	2.9 ± 0.04 ^*,£^
**CSCs 4 wk**	3.5 ± 0.02 ^*^	2.8 ± 0.34 ^*,£^	2.6 ± 0.09 ^*,£^
**CSCs 8 wk**	7.2 ± 0.25 ^*$,#^	3.5 ± 0.46 ^*,£^	2.6 ± 0.10 ^*,£,&^
	**Doubling Time (Day)**		
	Standard culture conditions	Adherent cells derived from CSCs cultured in DM for 10 days	Adherent cells derived from CSCs cultured in DM for ≥ 2 months
**P cells**	2 ± 0.25	/	/
**CSCs 2 wk**	3.6 ± 0.05 ^*^	2.9 ± 0.31 ^*,£^	2.9 ± 0.04 ^*,£^
**CSCs 4 wk**	3.5 ± 0.02 ^*^	2.8 ± 0.34 ^*,£^	2.6 ± 0.09 ^*,£^
**CSCs 8 wk**	7.2 ± 0.25 ^*$,#^	3.5 ± 0.46 0,^*£^	2.6 ± 0.10 ^*,£,&^
	**Doubling Time (Day)**		
	Standard culture conditions	Adherent cells derived from CSCs cultured in DM for 10 days	Adherent cells derived from CSCs cultured in DM for ≥ 2 months
**P cells**	2 ± 0.25	/	/
**CSCs 2 wk**	3.6 ± 0.05 ^*^	2.9 ± 0.31 ^*,£^	2.9 ± 0.04 ^*,£^
**CSCs 4 wk**	3.5 ± 0.02 ^*^	2.8 ± 0.34 ^*,£^	2.6 ± 0.09 ^*,£^
**CSCs 8 wk**	7.2 ± 0.25 ^*,$,#^	3.5 ± 0.46 ^*,£^	2.6 ± 0.10 ^*,£,&^

Viable cells were counted by Trypan Blue dye exclusion after 1, 4, 7, and 10 days. The doubling time was calculated using the formula T = (T_2_ - T_1_) × log 2/log (Q_2_/Q_1_), where: T_1_, day 0; T_2_, day 10; Q_1_, cell number at day 0; and Q_2_, cell number at day 10. In the first column, the doubling time is referred to cells grown in their standard culture conditions as reported in materials and method, i.e., differentiated-cell medium (DM) for P cells and stem-specific medium (SsM) for CSCs. In the second and third column, CSCs where grown in DM for maximum 10 days or at least 2 months, respectively. Values are the means ± SEM of four independent experiments. Statistical legend: *p* < 0.05 (*) CSCs or adherent cells derived from CSCs versus P cells; ($) CSCs 2 weeks versus CSCs 8 weeks; (#) CSCs 4 weeks versus CSCs 8 weeks; (£) adherent cells derived from CSCs versus the relative CSCs; (&) adherent cells derived from CSCs cultured in DM for 10 days versus the same cells cultured for more than 2 months.

**Table 2:** mRNA expression levels of stem, EMT, and quiescent markers in parental (P), cancer stem cells (CSCs), and adherent cells derived from CSCs (AdCSCs) and cultured in the differentiation medium for 10 days or more than 2 months. The data are shown as the average of fold change relatively to P cells ± standard error.

	CDH1 (fold change)	ZEB1 (fold change)	SOX2 (fold change)	NANOG (fold change)	OCT3/4 (fold change)	MAD2L1 (fold change)	cyclinB1 (fold change)
P	1	1	1	1	1	1	1
CSCs 2wk	2.32 ± 1.05	1.19 ± 0.28	4.64 ± 0.82	2.67 ± 0.04	2.20 ± 0.10	0.65 ± 0.33	0.31 ± 0.08
CSCs 4wk	1.65 ± 0.54	1.30 ± 0.58	11.85± 3.23	3.54 ± 1.50	2.28 ± 0.69	0.61 ± 0.12	0.69 ± 0.14
CSCs 8wk	0.17 ± 0.01	1.64 ± 0.02	24.42± 6.84	6.99 ± 2.71	3.12 ± 0.70	0.16 ± 0.02	0.14 ± 0.07
AdCSC 2w 10 days	1.12 ± 0.15	0.59± 0.09	0.42 ± 0.11	0.98 ± 0.11	1.70 ± 0.28	0.66 ± 0.11	1.01 ± 0.16
AdCSC 4w 10 days	1.56 ± 0.15	0.98 ± 0.03	0.56 ± 0.15	1.73± 0.46	2.01 ± 0.30	1.11 ± 0.32	0.94 ± 0.12
AdCSC 8w 10 days	1.29 ± 0.27	1.08 ± 0.04	0.30 ± 0.09	0.99 ± 0.18	1.83 ± 0.11	0.83 ± 0.13	0.61 ± 0.11
AdCSC 2w ≥2 months	1.92 ± 0.34	0.67 ± 0.12	0.14 ± 0.05	0.82 ± 0.14	1.53 ± 0.24	0.54 ± 0.06	0.94 ± 0.16
AdCSC 4w ≥2 months	2.75 ± 0.21	0.83 ± 0.11	0.50 ± 0.16	0.80 ± 0.32	2.23 ± 0.15	0.82 ± 0.16	0.86 ± 0.16
AdCSC 8w ≥2 months	2.73± 0.19	0.65 ± 0.06	0.64 ± 0.10	0.57 ± 0.19	1.35 ± 0.15	0.95 ± 0.17	1.07 ± 0.27

## Supplementary Materials

The following are available online at https://www.mdpi.com/2073-4409/9/7/1572/s1, Figure S1: (A) Bright field images of Panc1 cells cultured in the SsM for 1, 4, and 7 days. Scale bar: 100 μm. (B) Bright field images of PaCa44, PaCa3, and PC1J parental (P) cells and CSCs cultured for 8 weeks. Scale bar: 100 μm. C) Bright field representative images of Panc1 CSCs cultured in the stem-specific medium for 12 weeks. Scale bar: 100 μm. Figure S2: Average of the sphere diameters (A) and representative images (B) of cells grown for 30 days after the seeding of 1 cell/well of Panc1 P cells and CSCs. Scale bar: 100 μm. (C) Representative images of soft agar colony formation assay of Panc1 P and CSCs. D) Beta-galactosidase-based assay for the detection of senescent cells, which are indicated by arrows, in Panc1 positive control, P cells, and CSCs. Scale bar: 100 μm. Figure S3: Heatmap representation of the unsupervised hierarchical clustering of target gene expression across all samples based on their ΔCT values obtained through real-time PCR. Figure S4: Metabolomic Pathway Analysis (MetPA) generated by MetaboAnalyst software package by comparing (A) P cells versus CSCs 2 weeks; (B) CSCs 2 weeks versus CSCs 4 weeks; (C) CSCs 4 weeks versus CSCs 8 weeks. All the matched pathways are displayed as circles. The color of each circle is based on *p*-values (darker colors indicate more significant changes of metabolites in the corresponding pathway), whereas the size of the circle corresponds to the pathway impact score. Select pathways with high pathway impact and/or high p-value are labeled. Figure S5: qPCR analysis of Glut-1 transporter, phosphofructokinase (PFK), and enolase in Panc1 P cells and CSCs. The values are reported as fold change relative to P cells. Legend: white: parental (P) cells; light gray: CSCs 2 weeks; dark grey: CSCs 4 weeks; black: CSCs 8 weeks. Values are the means (± SE) of three independent biological replicates. Statistical legend: *p* < 0.05 (*) P cells versus CSCs; (#) CSCs 4 weeks versus CSCs 8 weeks. Table S1: Analysis of mRNA expression levels of stem markers (SOX2, NANOG, and OCT3/4) and of quiescence markers (MAD2L1, Cyclin B1, and RPLP0) in the three PDAC cell lines PaCa44, PaCa3, and PC1J cultured in the SsM for 4 and 8 weeks.
